# Neutralising antibodies against SARS-CoV-2 give important information on Covid-19 epidemic evolution in Rabat, Morocco, March 2020-February 2021

**DOI:** 10.4314/ahs.v23i3.46

**Published:** 2023-09

**Authors:** Nadia Touil, Charifa Drissi Touzani, El Mostafa Benaissa, Jalal Kasouati, Zineb Rhazzar, Hicham El Annaz, Nadia El Mrimar, Lamiae Neffah, Rachid Abi, Rida Tagajdid, Safae El Kochri, Mariette Ducatez, Youssouf Akhouad, Ahmed Reggad, Zouhour El Kassimi, Abdelhamid Zrara, Fatna Bssaibis, ElMostapha El Fahime, Idriss Lahlou Amine, Abdelkader Belmekki, Yashpal Singh Malik, Mostafa Elouennass, Khalid Ennibi

**Affiliations:** 1 Unité de Culture Cellulaire, Centre des Maldies Infectieuses et Tropicales, Hôpital Militaire d'Instruction Mohammed V de Rabat, Morocco; 2 Virologie Moléculaire Onco-Biologie, Faculté de Médecine et de Pharmacie, Université Mohammed V, Souissi-Rabat, Morocco; 3 Equipe de Reherche en Epidemiologie Bacterienne, Faculté de Médecine et de Pharmacie, Université Mohammed V, Souissi-Rabat, Morocco; 4 Laboratoire de Biostatistique, de Recherche Clinique et d'Epidémiologie Faculté de Médecine et de Pharmacie, Université Mohammed V, Souissi-Rabat, Morocco; 5 Centre de Transfusion Sanguine, Hôpital Militaire d'Instruction Mohammed V de Rabat, Rabat, Morocco; 6 Service de Virologie, Centre des Maldies Infectieuses et Tropicales, Hôpital Militaire d'Instruction Mohammed V de Rabat, Morocco; 7 IHAP, Université de Toulouse, INRAE, ENVT, Toulouse, France; 8 Plateforme fonctionnelle génomique, UATRS-CNRST, Rabat, Morocco; 9 Service d'Immunologie, Hôpital Militaire d'Instruction Mohammed V de Rabat, Morocco; 10 College of Animal Biotechnology, Guru Angad Dev Veterinary and Animal Science University (GADVASU), Punjab, India

**Keywords:** SARS-CoV-2, Serum neutralizing assay, Seroprevalence, Rabat, Morocco

## Abstract

**Background:**

The SARS-CoV-2 is an extremely contagious and acute viral disease mainly affecting humans.

**Objective:**

To estimate seroprevalence of SARS-CoV-2 neutralizing antibodies (NAbs) for illegible armed force individuals living in Rabat, Morocco

**Method:**

A convenience sample (N = 2662) was conducted from May 2020 to February 2021. We used the standard neutralization assay to quantify the NAbs titers. A serum was positive when the titer was 1:4. High positive NAbs titers were defined when ≥ 1:32.

**Results:**

Demographic and socioeconomic status did not affect seroprevalence data. An overall seroprevalence of 24,9% was found. Sera from blood donors, young recruits and auto-immune population had lower NAbs titers. However, titers were above 1:16 in 9% of the population with high risk of SARS-CoV-2 exposure. Seropositivity increased over time with values reaching peaks after the epidemic waves (2.4% in May 2020; 16.2% in August 2020; 22.7% in December 2020 and 37% in February 2021).

**Conclusion:**

And increase of NAbs was observed over time and correlated with the post-epidemic waves of COVID-19 in Morocco.

## Introduction

The Severe Acute Respiratory Syndrome Coronavirus 2 (SARS-CoV-2) is a highly contagious viral disease that mainly affects humans^1^. As of now, 263.5 million individuals had been exposed or infected with COVID-19^2^. In Morocco, the first confirmed case of SARS-CoV-2 was detected on March 2nd 2020. A few days later, human-to-human transmission of disease occurred and Moroccan's government announced a nationwide lockdown with end date to the lockdown measures was June 2020. During the fall season (November- December 2020), confirmed cases of COVID-19 spiked and high mortality rates varying between 70 and 90 deaths/day were recorded. The control of asymptomatic carriers through mass-testing or the contact-tracing strategies to slow down the COVID-19 epidemic looks implausible.

To gain some insights on the proportion of asymptomatic individuals who might recover from COVI-19, screening of antibodies (Abs) against SARS-CoV-2 is essential in large populations with sensitive and specific serological testing. However, for immune responses, neutralizing antibodies (NAbs) tests are preferred to non-neutralizing Abs assays^3^,^4^ and for, their detection, Lau and Co-authors5 recommended the use of live virus. Only a few studies have reported NAbs based on authentic SARS-CoV-2^2^,^5^.

Within the last year there have been several seroprevalence studies that have shown inconsistencies between the immunity level in SARS-CoV-2 infected individuals. In this study we aimed to inform estimates of seroprevalence of neutralizing SARS-CoV-2 NAbs to COVID-19 among 2662 individuals using the microneutralization assay.

## Materials and Methods

### Study design and Setting

The study was performed at the Center of Virology and Infectious Tropical diseases (CVMIT), Military Hospital of Rabat, Morocco. The study was approved by the Institutional Ethical Review Committee. Oral or written consent were obtained from each participant and consent for children was obtained from the parents.

We used a convenience sampling to estimate SARS-CoV-2 seroprevalence for eligible service members, military families and army personnel (heath care workers and blood donors) residing in the district of Rabat. There were no criteria required to be a part of this sample except the consent of participants. Our study was partnered with the screening and testing for COVID-19 in the military hospital for all participants.

Critical information and data included sex, gender, socio-economic strata and information of previous COVID-19 infection if known.

In addition, our sampling incorporated military families at-risk of auto-immune diseases (ADs) and other civilian employees and contractors from different regions.

### Sample collection

Blood samples were taken along with nasopharyngeal swabs from consenting persons between May 2020 and 1^st^ March 2021. They were transported to the Cell Culture Unit, CVMIT, Military Hospital of Rabat for analysis.

### Viral neutralization assay

The assay was performed at high biosafety level containment laboratory (BSL-3) in the CVMIT, Military Hospital of Rabat.

### Serum neutralisation assay

Both Vero E6 (ATCC CRL-1586) and Vero CCL-81 cell lines were maintained in DMEM supplemented-inactivated 8% Foetal bovine serum (FBS). hCoV-19/Morocco/HMIMV_279CC/2020 (EPI_ISL_971451) virus was isolated from an oropharyngeal swab specimen using Vero E6 cells as described6 and further adapted on Vero cells in four passages (P4). Virus titration was performed using 50% tissue culture infective dose (TCID^50^) assay.

The neutralisation test was carried out as described previously^7^ with some modifications. Briefly, sera were heat-inactivated for 30 min at 56°C and processed as following: 50 µL of serum-free DMEM medium was added into columns 1–12 of a 96-well tissue culture plate, and then 50 µL of each serum was pipetted into column 1 in four replicates in 4 twofold serial dilutions (starting 1:4). Tips were replaced between dilutions. Then, 300 TCID^50^ (0.01 MOI) of virus per 50 µL was added to each well. After incubation at 37°C with 5% CO2 for 2 h, Vero cells in DMEM containing 2% FBS were added to the wells at a concentration of 3 × 10^4^ cells/well. Cells were grown for 48-72 hours and the cytopathic effect (CPE) were observed under light microscopy.

The highest serum dilution inhibiting at least 50% of the CPE was indicated as the neutralization titer and samples with NAbs titers of ≥4 were considered positive^7^.

To standardize inter-assay procedures, negative and positive controls were prepared synchronously with positive control samples showing high (1: ≥32) and low (1:4) neutralizing activity were included in each assay session. In parallel, a back titration plate was included to control the effective infectious titer of the virus.

### Assay specificity and sensitivity

A panel of pre-pandemic sera (n= 14 including 6 samples from patients with chronic inflammatory diseases) was used as negative controls to validate the specificity of our assay.

Positive controls were obtained from the laboratory of Virology, CVMIT with known neutralization titers (NAbs titers: from 4 to ≥32). These positive serums were collected from blood samples from 56 health care workers and recovered from COVID-19. The diagnosis of COVID-19 in these patients was based on positive RNA test for SARS-CoV-2 from nasopharyngeal swab samples before their hospitalization. 7 patients had a median NAb titer of 1:4; 41 individuals had titers between 8-16 and 11 persons had NAbs tietrs more than 1:32. NAbs were not detected in one asymptomatic patient who quickly cleared the virus. Titers were higher in patients with symptomatic infection (p = 0.02) and are positively correlated with severity of COVID-19 pneumonia (unpublished data).

### Data analysis

Data entry of all questionnaires was performed using Microsoft Excel 2013 software. Statistical analysis was realised using the SPSS software version 25. Comparison between sampling periods was done using the Chi-Square test. A p value <0.05 is considered statistically significant.

## Results

We collected prospectively 2662 blood samples between 0 and 84 years of age. Our cohorts included blood donors (n=489), employees in military manufacturing (n=82), healthcare professionals from the CVMIT (n=164), young recruits (n=677), a population with suspicious autoimmune diseases (ADs) (n= 653) and other participants from general population coming from all over the country for COVID-19 diagnosis during the period of February 2021 (n= 597) (Table 1). Information of age in different subgroups is given in Table 1. Age was normally distributed between groups with a median value of 42 years old.

**Table:** Table 1

n (%)	Age (n)	Sex (n)		Seroprevalence	Seronegative	Seropositive, n (%)			

		Male	Female	n (%)		n (%)	NAbs titres 8-16	NAbs titres ≥32
All participants								
	2662	42 (0-84)	1732	895	662 (24.9)	2000 (75.1)	603 (91.0)	59 (9.0)
Blood donors	489 (18.3)	42 (22-62)	485	4	108 (16.3)	381 (19.0)	108 (100)	0 (0.0)
Health care workers	164 (6.1)	42 (22-62)	87	77	74 (11.2)	90 (4.5)	49 (66.2)	25 (33.8)
young recruits	677 (25.4)	21.5 (21-22)	418	245	116 (17.5)	561 (28.0)	116 (100)	0 (0.0)
Manufacturing								
workers	82 (3.0)	45 (23-67)	52	30	28 (4.2)	54 (2.7)	17 (60.7)	11 (34.1)
Auto-immune								
population	653 (24.5)	41 (0-83*)	261	373	80 (12.0)	573 (28.6)	80 (100)	0 (0.0)
Other participants	597 (22.4)	47 (21-74)	429	166	256 (38.6)	341 (17.0)	233 (91.0)	23 (9.0)
p value		***0.062***		***0.074***				

A relatively high overall seroprevalence in the present study was noticed. The rate of seroprevalence infection with SARS-CoV-2 reached 24,9% (662/2662) over all the study period. 91% of seropositive sera had Nabs titers less than 1:16. The seroprevalence was independent of age and sex and no correlation of NAb titers with rank was deciphered. Males constitute the 2/3 of the population. Group individuals younger than 17 years and older than 74 years constitute 3.8% (n=101) and 2.6% (n=122) respectively. These groups were vulnerable and might have ADs.

Participants did not report previous infection of SARS-CoV-2 except for the health care workers who were routinely diagnosed for COVID-19. In the high-risk patient groups 6 and 7 patients among health care and manufacturing workers experienced COVID-19 symptoms. They were confirmed tested SARS-Cov-2 positive by RT-PCR. 100% were symptomatic with mild to severe disease consisting of cough, fever and sore throat. They were close contacts of a confirmed case and show a high prevalence of infection (45% of them had contact with the virus and 34% had higher NAbs titers; 34% of workers in the manufactures were positive and 39% had titers more than 1:32) as shown in Table 1. These titers were higher than those observed in the blood donors, young recruits and ADs individuals (a median NAbs titer of 1:15 was obtained).

The evolution of SARS-CoV-2 infection over the study period is given in Figure 1. An overall increase of NAbs was observed over time. In the first survey (May-June 2020), an infection rate of 4.3% was found. In the second period in August 2020, the prevalence of NAbs increased and reached a median value of 16.2%. NAbs seroprevalence went down in September 2020 (7.6%), after which, it increased to 22.7% and stabilised during the following month (November 2020). In December 2020 and February 2021, almost 37% of participants had NAbs (Figure 1).

**Figure:**
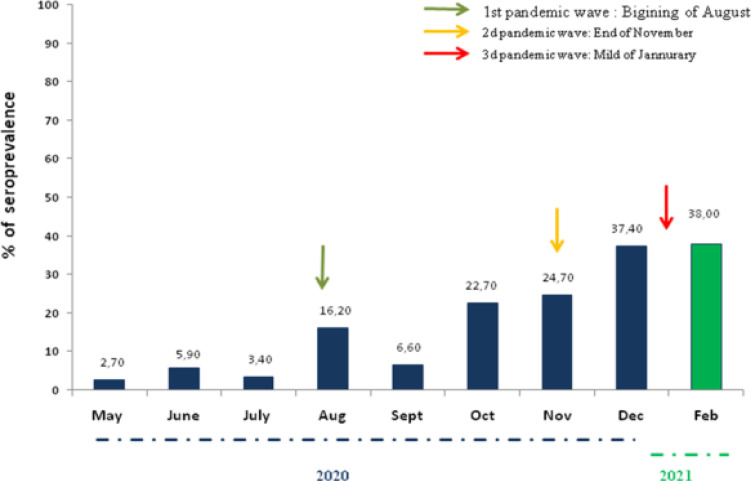
Figure 1

Time of blood collection for NAb analysis influenced the NAb titers. There was a statistically significant differences in median NAb titers observed between the sampling period with a significant higher level in August 2020, October 2020, December 2020 and February (p<0.001). Lower NAb titers were found in participants in May-July 2020 and September 2020.

## Discussion

NAbs testing is an important tool for public health authorities to better estimate the epidemic evolution and to evaluate the effectiveness of control measures8,9. We did not have access to the general target population for a probability sampling due to quarantine and mobility restrictions imposed by our government. Therefore, our results may not be extrapolated to the general population. Meanwhile, our findings provide important information about SARS-CoV-2 circulation in the region of Rabat during periods of lockdown and lifting containment measures from March 2020 to February 2021, such studies are rare.

In addition, in the context of our study, a large sample of individuals were identified using the gold standard assay with 100% of specificity and 98.25% of sensitivity. This test was developed during April 2020 within the project sponsored by the Ministry of Higher Education and innovation and the National Scientific Consortium for Biomedical Research and Innovation to substitute those commercial IVD serological tests which could not be easily available at the time even in the most developed region.

Morocco imposed a total lockdown from March to June 2020 soon after the surge of SARS-COV-2 infection in the country^10^. During this period of time, the seropositive rates were very low and not different from that found in Italy (3.4% versus 2.6%) translating that the imposed control measures were efficient to slow the spread of COVID-19^11^. However, these rates remain higher than the prevalence estimated in Icelanders (3.4% versus 0.9% tested positive).^12^

From July 2020, our country started to lift restrictions leading to a surge of SARS-CoV-2 infected cases in many regions with higher mortality and morbidity rates^10^. This was also the case in November 2020 leading to a new lockdown in the entire country. This high infection rate in these cycles may explain in part the higher levels of NAbs rates in December 202010. Our estimate of the NAbs titers is higher than estimates described in Oman using the live serum neutralisation assay where 8.5-16.8% sera tested positive in October -December 2020^8^, but lower than those found between February–March 2021 (36% versus 83.7%). This peak began in January 2021 as the transmittable alpha variant began to circulate worldwide. This factor may explain in part, the highest proportion of seroprevalence (38%) observed in the group of individuals who have been in pre-symptomatic stages of the disease or who had been infected with SARS-CoV-2 but not declared. Thus, an estimate of seroprevalence in the underlying population might be true.

A high seroprevalence of asymptomatic SARS-CoV-2 infection in blood donors, young recruit's army personnel and in the sub-group of individuals with suspected ADs was found and almost one person over 7 had contact with the virus in a population (n=1870). Interestingly, these infected persons had NAbs less than 1:16, a titer which we consider very low as compared to that obtained in mild or severely infected individuals (NAbs more than: 32 for our positive controls or in patients who experienced severe dieseas^8^.

A high rate of symptomatic infection was found in the high-risk group (heath care and manufacturing workers), as it has been observed in Lebanon^8^ and in Ireland^12^. All workers have safety measures inside their work places and the employers provided personal protective equipment and testing for every employee after an outbreak. Our results corroborate data published in similar cohorts with known general factors in this cohorts^13^.

In conclusion, our study while not conducted on a probalistic sampling to estimate the true burden of infection in Morocco, has helped Moroccan government to contain the high contagiousness and rapid spread of SARS-CoV-2. The progressive increase in the prevalence of NAbs over the study period coincided with the most important surges in SARS-CoV-2 infections and deaths as has been described elswere^8^.

## Author agreement

All authors have seen and approved the final version of the manuscript being submitted. The article is the authors' original work, has not received prior publication, and is not under consideration for publication elsewhere.
